# Pregnancy Outcomes among Obese Pregnant Women with Varying Levels of Vitamin D in King Abdulaziz University Hospital: A Single-center Retrospective Study

**DOI:** 10.7759/cureus.6220

**Published:** 2019-11-23

**Authors:** Anas M Fallatah, Areej J Bahrawi, Hussam Babatin, Khalid M Nassibi, Yousef AlEdreesi, Hassan S Abduljabbar

**Affiliations:** 1 Medicine, King Abdulaziz University, Jeddah, SAU; 2 Obstetrics and Gynecology, King Abdulaziz University, Jeddah, SAU

**Keywords:** vitamin d, maternal, neonatal, pregnancy, obese, bmi, outcomes

## Abstract

Background

Vitamin D deficiency among pregnant women is a global issue. Despite its high prevalence, the optimal level of vitamin D among pregnant women is not well established. On the other hand, multiple adverse pregnancy outcomes have been strongly associated with vitamin D deficiency.

Objectives

To identify the potential effect varying levels of vitamin D have on maternal and neonatal outcomes.

Methods

This is a non-intervention retrospective record review conducted on pregnant women who delivered in King Abdulaziz University Hospital, Jeddah, Saudi Arabia between January 1, 2013, and December 31, 2018. Data were collected from their hospital electronic files and analyzed by Statistical Package for Social Sciences (SPSS; IBM Corp., Armonk, NY) version 24. A p-value of <0.05 is used to calculate statistical significance.

Results

A total of 9095 pregnant women had delivered in the last five years, 137 of these pregnant women had vitamin D measurement during their pregnancy. Out of 137, 99 were deficient, 20 optimal, 11 therapeutic, and two excess. A total of 97 (70.8%) were Saudis and 40 (29.2%) were non-Saudis. The majority of pregnant women in obese class 1 and 2 were deficient with 99 cases, while obese class 3 was mostly those with optimal levels. Regarding pregnancy outcomes, those with vitamin D deficiency had the majority of undesired antepartum, neonatal, and postpartum outcomes. Placenta previa, endometritis, poor APGAR scores, birth defects, intrauterine fetal demise, low birthweight, and macrosomia were significantly associated with abnormal vitamin D levels (P < 0.05).

Conclusion

Although vitamin D optimum level during pregnancy is not known, pregnant women with deficient levels appeared to have more serious risks to develop adverse pregnancy outcomes. Therefore, early screening during prenatal visit or antenatal for vitamin D level with vitamin D supplementations is important to reduce these negative pregnancy outcomes for pregnant women with deficient levels.

## Introduction

Vitamin D is the most micronutrient that has gained and sustained massive interest in the fields of health and biomedical research community [1]. Vitamin D receptors are expressed pervasively in the entire of human body’s cells which indicates an involvement of vitamin D-mediated effects on decisive metabolic networks [2]. Vitamin D deficiency is defined as 25(OH) D level to be lower than 50 nmol/l (20 ng/ml). In addition, it is an epidemic and it became to be a global issue [3]. Locally, The Middle East and North African (MENA) region has a tremendous prevalence of vitamin D deficiency in comparison to global studies and it is mainly seen in women of varying ages [4]. In Saudi Arabia, according to meta-analysis study made in 2016, it showed that almost 81% of Saudis are considered to be deficient [1]. Additionally, this pattern is also seen in other Arabian Gulf countries [5-8].

Initially, it was believed that deficiency of vitamin D only manifests on bone metabolism, yet now it has become more evident that it has a wide range of extra-skeletal effects such as: neuropsychiatric disorders, deterioration of immune system, increased risk in cardiovascular disease and hypertension, and at last but not least increased risk of mortality [9].

Moreover, race has found to play a major role. African-American adults have been considered to be important risk factor for vitamin D deficiency having the highest prevalence rate of vitamin D deficiency. Other risk factors such as obesity, lack of college education, and lack of daily milk consumption were also identified for vitamin D deficiency [10].

Vitamin D deficiency in pregnant women is common in many parts of the world, and there is a very strong correlation between vitamin D deficiency and multiple adverse pregnancy outcomes [11]. However, the role and metabolism of vitamin D in the pregnancy is not well known [11-14]. In spite of the reported high prevalence of deficiency and the possible consequences, the desired optimal level needed for pregnant women in their body and the amount of vitamin D intake required to maintain adequate levels is not very well documented [11,14-17].

Despite all these studies none of them were done in the western region of Saudi Arabia which encourages us to conduct our research. This study aims to assess the pregnancy outcomes among obese vitamin D deficient pregnant women in King Abdulaziz University Hospital, Jeddah, Saudi Arabia.

## Materials and methods

This is a retrospective record review study carried out over five years period from January 2013 to December 2018. The study was conducted among obese pregnant women attending King Abdulaziz University Hospital (KAUH), Jeddah, KSA. These electronic medical file records were obtained from the hospital’s Medical Records Department. Pregnant women with BMI less than 30 kg/m^2^ and missing vitamin D levels were excluded. Out of 1037, 137 patients were found to have vitamin D measurement during their pregnancy and were involved in the study for further analysis. Ethical approval to conduct the study was obtained from KAUH Institutional Review Board with reference number 78-19. Demographic variables such as nationality, gender, age, height, weight, BMI were taken into consideration. Vitamin D metabolites serum concentration was determined by radioimmunoassay kits from Immunodiagnostic Systems Ltd, Boldon, UK. A trained hospital staff (phlebotomist) collected venous blood samples, separated serum, and stored at -70°C until analysis. The treated samples were then assayed using competitive-binding radioimmunoassay. Subjects were classified into five categories: (1) vitamin D deficiency, 25(OH)D <29 ng/mL; (2) adequate, 25(OH)D 30-39 ng/mL; (3) optimal, 25(OH)D 40-59 ng/mL; (4) therapeutic level, between 30 and 80 ng/mL; and (5) excess level, greater than 100, which is consistent with other studies.

Adverse pregnancy outcomes

Adverse antepartum variables included the following: Pregnancy-induced hypertension (PTH), preeclampsia, antiphospholipid syndrome (APS), impaired glucose tolerance test (GTT), gestational diabetes mellitus (GDM), pre-premature rupture of membrane (PPROM), premature rupture of membrane (PROM), antepartum hemorrhage (abruptio placenta, placenta previa, placenta accrete and low lying placenta), and maternal mortality; fetomaternal complications (oligohydramnios, polyhydramnios, placental hematoma, and cord prolapse), preterm labor (PTL), induction of labor, mode of delivery (spontaneous vaginal delivery (SVD) or cesarean section (CS)), type of CS (emergency or elective), failure to progress (FTP), cephalopelvic disproportion (CPD), rate of cesarean section, anemia, and urinary tract infection (UTI).

Adverse postpartum variables included the following: Postpartum hemorrhage (PPH), endometritis, vaginal laceration, perianal laceration 1st, 2nd, 3rd degree, internal hemorrhoids, surgical site infection (SSI), and wound dehiscence.

Adverse neonatal outcomes included the following: Gestational age, birth weight, fetal sex, fetal presentation, APGAR score at 1 and 5 minutes, admission to neonatal intensive care unit (NICU), birth defects and injuries, intrauterine fetal demise (IUFD), stillbirth, and neonatal death.

We used the Statistical Package for Social Sciences (SPSS; Release 23.0.0.0, IBM Corp., Armonk, NY). Analysis of data was conducted using SPSS for Windows software. Demographic, antepartum, and postpartum data were examined. The independent t-test and correlation tests were used for comparison of quantitative variables. The chi-square test was used as a test of significance for comparison of categorical variables. P ≤ .05 was chosen as the level of statistical significance.

## Results

The aim of the study is to identify the potential effect varying levels of vitamin D have on maternal and neonatal outcomes. It included 137 pregnant women who delivered their newborn by cesarean section and those which had a BMI measurement of more than 30. Most of the pregnant women were Saudis (97; 70.8%) while non-Saudis only represented 40 (29.2%). Table 1 demonstrates the socio-demography of the pregnant women. A total of 78.6% of pregnant women with obese class I had vitamin D deficiency, 22 (16.7%) of women with obese class II had a deficiency in vitamin D, and none was seen among obese class III, which appeared to be statistically significant with undesired pregnancy outcomes (P < 0.05). Most of vitamin D deficiencies occurred in pregnant women between the ages of 30 and 34 (29.5%). Women with multigravida exhibited higher rate of vitamin D deficiency (71.8%) compared to primigravida. Almost 6.8% of all preterm neonates had mothers with vitamin D deficiency.

**Table 1:** Sociodemographic and maternal characteristics of the studied pregnant women in relation to vitamin D levels (n = 137) ^a ^P < 0.05 SA: Saudis; N-SA: Non-Saudis; GA: Gestational age.

		Vitamin D levels								
		Deficient		Optimal		Therapeutic		Excess		
		Count	Table N %	Count	Table N %	Count	Table N %	Count	Table N %	
Nationality	SA	71	53.8%	12	9.1%	9	6.8%	2	1.5%
	N-SA	28	21.2%	8	6.1%	2	1.5%	0	0.0%	
Age^a^	<30	23	17.4%	3	2.3%	1	0.8%	0	0.0%
	30-34	39	29.5%	4	3.0%	4	3.0%	0	0.0%	
	35-39	25	18.9%	9	6.8%	4	3.0%	2	1.5%	
	40-44	12	9.1%	3	2.3%	2	1.5%	0	0.0%	
	45-50	0	0.0%	1	0.8%	0	0.0%	0	0.0%	
BMI Group^a^	Obese Class I	77	58.3%	13	9.8%	7	5.3%	1	0.8%
	Obese Class II	22	16.7%	5	3.8%	4	3.0%	1	0.8%	
	Obese Class III	0	0.0%	2	1.5%	0	0.0%	0	0.0%	
Gravidity	Primigravida	4	3.1%	3	2.3%	0	0.0%	0	0.0%
	Multigravida	94	71.8%	17	13.0%	11	8.4%	2	1.5%	
Parity	Nullipara	5	3.8%	3	2.3%	0	0.0%	0	0.0%
	Multipara	93	71.0%	17	13.0%	11	8.4%	2	1.5%	
GA	Preterm	9	6.8%	3	2.3%	2	1.5%	0	0.0%
	Full term	89	67.4%	17	12.9%	9	6.8%	2	1.5%	
	Post term	1	0.8%	0	0.0%	0	0.0%	0	0.0%	

Maternal and obstetrics characteristics of pregnant woman with complications such as anemia, preeclampsia, and gestational diabetes mellitus are demonstrated in Table 2. Anemia (71.1% vs 28.9%) and GDM (54.2% vs 45.8%) was more frequent in Saudi pregnant women than in non-Saudi pregnant woman. One-third of women between the ages of 35 and 39 developed gestational diabetes mellitus. Anemia occurred most frequently to woman between the ages of 30 and 34 (37.8%). Furthermore, only two patients who suffered severe preeclampsia were Saudi, and two out of the three patients who developed mild preeclampsia were non-Saudi. Urinary tract infections in Saudi women were higher than in non-Saudi woman during pregnancy. Also, patients with obese class I had higher rates of UTIs (68.3%), GDM (66.7%), and anemia (70%) compared to other groups. Multigravida pregnant women exhibited higher rates of anemia (94.4%), GDM (95.8%), and UTIs (92.7%) compared to primigravida. More than half of the females with GDM underwent an elective cesarean section.

**Table 2:** Maternal and obstetrics characteristics with complications such as anemia, preeclampsia, gestational diabetes mellitus (GDM), and urinary tract infections (n = 137) ^a ^P < 0.05 GDM: Gestational diabetes mellitus; UTIs: Urinary tract infections; SA: Saudis; N-SA: Non-Saudis.

		Mild Preeclampsia		Severe Preeclampsia		Anemia		GDM		UTIs	
Nationality	SA	1	33.3%	2	100.0%	64	71.1%	13	54.2%	31	75.6%
	N-SA	2	66.7%	0	0.0%	26	28.9%	11	45.8%	10	24.4%
BMI^a^	Obese Class I	1	33.3%	0	0.0%	63	70.0%	16	66.7%	28	68.3%
	Obese Class II	2	66.7%	2	100.0%	25	27.8%	8	33.3%	12	29.3%
	Obese Class III	0	0.0%	0	0.0%	2	2.2%	0	0.0%	1	2.4%
Age	<30	1	33.3%	0	0.0%	14	15.6%	4	16.7%	10	24.4%
	30-34	1	33.3%	0	0.0%	34	37.8%	5	20.8%	13	31.7%
	35-39	1	33.3%	1	50.0%	28	31.1%	8	33.3%	10	24.4%
	40-44	0	0.0%	1	50.0%	14	15.6%	7	29.2%	8	19.5%
	45-50	0	0.0%	0	0.0%	0	0.0%	0	0.0%	0	0.0%
Parity	Nulliparous	0	0.0%	0	0.0%	5	5.6%	1	4.2%	3	7.3%
	Multipara	3	100.0%	2	100.0%	85	94.4%	23	95.8%	38	92.7%
Gravidity	Prim gravida	0	0.0%	0	0.0%	5	5.6%	1	4.2%	3	7.3%
	Multigravida	3	100.0%	2	100.0%	85	94.4%	23	95.8%	38	92.7%

Table 3 shows the vitamin D levels in pregnant women with maternal morbidity. Vitamin D deficiency was higher in pregnant women with anemia (49.2%). However, vitamin D deficiency was found in only 12.1% females with GDM (3%) with mild preeclampsia, and 2% with severe preeclampsia. While the most prevalent placental pathology among our sample was placenta previa, which was more commonly seen among pregnant women with deficient vitamin D, which showed a significant association (P < 0.05). One case of PTL was also seen in a pregnant woman with vitamin D deficiency. Moreover, there was also one case seen in a pregnant woman with therapeutic levels of vitamin D, which showed significant association (P < 0.05). A total of 11 cases had induction to terminate their pregnancy, most of them were also vitamin D deficient. Surprisingly, SSI appeared to be seen only in pregnant women with deficient vitamin D levels. Regarding UTI, it was also highly seen among pregnant women with vitamin D deficiency, with 31 cases.

**Table 3:** Vitamin D levels in pregnant women with different maternal complications ^a ^P < 0.05 GDM: Gestational diabetes mellitus; PIH: Pregnancy-induced hypertension; PROM: Premature rupture of membranes; PPH: Postpartum hemorrhage; SSI: Surgical site infection; UTI: Urinary tract infection.

	Vitamin D Levels			
	Deficient (n = 99)	Optimal (n = 20)	Therapeutic (n = 11)	Excess (n = 2)
Mild Preeclampsia	3 (3%)	0	0	0
Severe Preeclampsia	2 (2%)	0	0	0
GDM	16 (12.1%)	3 (15%)	3 (27.3%)	1 (50%)
PIH	1 (1%)	2 (10%)	0	0
PROM	0	0	0	0
Placenta Previa^a^	4 (4%)	0	0	0
Abruptio Placenta	1 (1%)	0	0	0
Low Lying Placenta	0	0	0	0
Placenta Accreta	1 (1%)	0	0	0
Maternal Mortality	0	0	0	0
Preterm Labor	1 (1%)	0	0	0
Induction of Labor	7 (7.1%)	2 (10%)	1	1
PPH	0	0	0	0
Endometritis^a^	0	0	1	0
SSI	3 (3%)	0	0	0
Anemia	65 (65.7%)	12 (60%)	8 (72.7%)	2 (100%)
UTI	31 (31.3%)	4 (20%)	3 (27.3%)	1 (50%)

Table 4 displays frequency of maternal comorbidities. In the observed sample, 19.7% of the studied pregnant women have comorbidities, of those 14.4% had vitamin D deficiency. Hypothyroidism was the most prevalent comorbidity in the sample, representing 8% of all comorbidities. Hypertension (HTN) was the second most common comorbidity representing only 4.4%. While diabetes mellitus type 1 and type 2 had been represented with three and two cases, respectively.

**Table 4:** Frequency of morbidity among the studied pregnant females with varying vitamin D levels (n = 137) HTN: Hypertension; DM: Diabetes mellitus; SLE: Systemic lupus erythematosus.

	Vitamin D Levels				
	Deficient	Optimal	Therapeutic	Excess	
Maternal Comorbidity	22	4	4	1
HTN	5 (22.7%)	0	1 (25%)	0
DM Type 1	2 (9.1%)	0	0	0
DM Type 2	3 (13.6%)	0	1 (25%)	0
SLE	1 (4.5%)	0	0	0
Hypothyroidism	6 (27.2%)	3 (75%)	1 (25%)	1 (100%)
Neurological Disease	1 (4.5%)	0	0	0
Psychiatric illness	2 (9.1%)	0	0	0
Bronchial Asthma	1 (4.5%)	0	1 (25%)	0
Hyperlipidemia	0	1 (25%)	0	0
Rheumatic Heart Disease	1 (4.5%)	0	0	0

Regrading neonatal outcomes from Table 5, most of the neonates who had low birthweight born to mothers with a deficient vitamin D level in comparison to optimal, therapeutic and excess levels. Yet, more than half of neonates born to vitamin D deficient mothers had normal birth weight. Both of these findings had significant association with p-value less than 0.001. On the other hand, poor Apgar scores at 1 minute were higher in women with vitamin D deficiency (13 cases), while those with optimal and therapeutic levels had less frequent events with 2 and 3, respectively. These results were also statistically significant with p-value less than 0.001. In addition, 8% of neonates were admitted to newborn intensive care unit (NICU) and most of them were related to mothers with deficient vitamin D levels. One case of IUFD was found in a pregnant woman with therapeutic levels of vitamin D, which is also statistically significant with p-value less than 0.001. Surprisingly, there is no event of oligohydramnios or polyhydramnios in our sample.

**Table 5:** Frequency of adverse neonatal outcomes among our sample (n = 137) ^a ^P < 0.05 ^b ^P < 0.05 IUFD: Intrauterine fetal demise; NICU: Neonatal intensive care unit.

		Vitamin D Levels			
		Deficient	Optimal	Therapeutic	Excess
Birthweight^b^	Low Birthweight	11	3	1	0
		11.2%	16.7%	9.1%	0.0%
	Normal Birthweight	84	14	10	2
		85.7%	77.8%	90.9%	100%
	Macrosomia	3	1	0	0
		3.1%	5.6%	0.0%	0.0%
Poor APGAR at 1 min^b^		13 (13.3%)	2 (11.1%)	3 (27.3%)	0
Poor APGAR at 5 min^b^		0	0	0	0
Birth Defects^a^		2 (2%)	0	1 (9.1%)	0
IUFD^a^		0	0	1 (9.1%)	0
NICU Admission		8 (8.1%)	2 (10%)	1 (9.1%)	0
Oligohydramnios		0	0	0	0
Polyhydramnios		0	0	0	0

From Table 6, we can see the most frequent fetal presentation is cephalic (82.3%) unlike breech which was only 12.4%. Most fetuses in the study were female (51.8%) with male estimated at 48.2%.

**Table 6:** Frequency of fetal characteristics

		Count	Percentage
Fetal Sex	Male	66	48.2%
	Female	71	51.8%
Fetal Presentation	Cephalic	79	82.3%
	Breech	17	17.7%

## Discussion

The findings of this study have displayed the widespread presence of vitamin D deficiency among obese pregnant women in KAUH, Jeddah. From our study, its prevalence was most marked in those who are obese class 1, whereas a lower prevalence was found in those who are obese class 2. A study conducted in Saudi Arabia and directed towards vitamin D deficiency among pregnant Saudi women displayed similar results [18]. Most of the study participants were either overweight or obese with the majority of pregnant women (91.9%) having vitamin D deficiency. On the contrary, a study conducted in Southern China showed the prevalence of vitamin D deficiency to be at 18.9%, with no significant differences in most adverse pregnancy outcomes [19].

A systematic review and meta-analysis confirmed the association between obesity and vitamin D deficiency within different age groups [20]. They also showed higher prevalence of vitamin D deficiency was 35% higher in obese subjects compared to non-obese subjects. Although, the study’s focus was not directed towards pregnant women, the clear effect of obesity on vitamin D deficiency was noted. Another recent study conducted in Spain, reported vitamin D deficiency in a high percentage of obese and overweight northern Spanish pregnant women (34.5%) [21]. It can be observed that the previously mentioned studies have had similar findings to ours.

According to a recent study conducted in India, complications including gestational diabetes, preeclampsia, and preterm labor were associated with vitamin D deficiency in pregnant women, which supports our study result that has also highlighted the complications at risk in pregnant obese women with different vitamin D levels [22]. Complications included anemia, gestational diabetes mellitus, preeclampsia, and urinary tract infections to name a few. On the other hand, those with excess levels of vitamin D were associated with GDM, and preterm labor according to a recent study [19]. These complications came to support our results, which showed pregnant women with excess levels of vitamin D developed GDM, induction of labor, anemia, and UTI.

The aforementioned study also displayed low birth weight and poor Apgar score in the neonates born to the mothers, which we also found regarding neonates of vitamin D deficient mothers that displayed a number of complications including low birth weight, macrosomia, poor APGAR scores at 1 min, and admission to the NICU although more than half had normal birthweight [19].

Another study, we also took note of, was a study conducted on the effects of vitamin D deficiency in pregnancy which states that effects of vitamin D may occur earlier in pregnancy than previously appreciated. And that to date, most studies regarding this topic focus on vitamin D deficiency in later stages of pregnancy [23].

A study conducted in the USA showed pregnant women with optimal levels of vitamin D were protected against adverse pregnancy outcomes with 57% lower risk than those with vitamin D deficiency [24]. Yet, our results showed those pregnant women with optimal levels of vitamin D developed GDM, pregnancy-induced hypertension (PIH), induction of labor, anemia, and UTI. Not only that, they also had low birth weight, macrosomia, poor APGAR scores, and NICU admission rates. Although these results were only seen in small portion of those with optimal vitamin D levels in comparison to vitamin D deficient but the relation remains unclear.

As for pregnant women with therapeutic levels of vitamin D, similar complications of those with optimal and deficient levels of vitamin D were present with the exception of PIH. All except for GDM were displayed in lower rates when compared to pregnant women with deficient and optimal levels of vitamin D. Regarding the neonates, low birth weight and NICU admission were found but similarly in low rates. Poor Apgar at 1 min was higher than in neonates born to mothers with optimal levels of vitamin D. Compared to a recent systemic review conducted from 13 randomized controlled trials (RCTs) it showed that incidences of preeclampsia, GDM, SGA, low birth weight, preterm birth, and cesarean section were not influenced by vitamin D status [25].

The WHO states that vitamin D supplementation improves maternal vitamin D status and may reduce the risk of pre-eclampsia, low birth weight and preterm birth. However, the data regarding the direct benefits and harms of supplementation is fairly limited. Therefore, the WHO does not recommend vitamin D supplementation to improve maternal and perinatal outcomes. This supersedes the previous recommendation in 2012 [25]. Although a study from the American Society for Bone and Mineral Research addresses that there was no harm from implementing vitamin D supplementation or screening for all pregnant women [26].

The following is enlisted in the American College of Obstetricians and Gynecologists: At this time there is insufficient evidence to support a recommendation for screening all pregnant women for vitamin D deficiency. However, we may consider screening for pregnant women thought to be at risk. 25(OH) D is used for screening and should be interpreted in the context of the individual circumstance. Most experts would agree that supplementing a 1000 to 2500 international units is safe. Higher dose regimens during pregnancy have not yet been studied [27]. In conclusion, vitamin D status seems to contribute to pregnancy outcome on both the maternal and neonatal level.

As what is usually the case, the study could have benefited from a larger sample. But then again as the title implies it is a single center study and hopefully other local centers could make use of the data if they wish to conduct a similar study. Also, in contrast to other global studies, our sample is probably more prone to vitamin D deficiency owing to the relatively less sun exposure, which is also observed in studies in other countries with similar lifestyle such as Iran and other Middle East countries, which in turn would lead to a higher prevalence of vitamin D deficiency possibly having an impact on the results. A similar relationship can be observed but in an inversely manner in the study from Southern China, which demonstrated a much lower prevalence and no significant difference in outcome.

## Conclusions

As our study showed, obese pregnant women with vitamin D deficiency are more prone to adverse maternal and neonatal outcomes. Due to the high prevalence of vitamin D deficiency in our region, an early screening during preconception period or first antenatal visit is vital. As we mentioned earlier, those who are deficient are going to benefit from vitamin D supplementation. At the end, we again recommend a population-based multi-center cohort study to provide more accurate knowledge about prevalence and impact not only of vitamin D deficiency but also other varying levels on pregnancy outcomes.

## Appendices

Author’s contributions:

Each author gave substantial contribution to the conception or design of the work and in the acquisition, analysis and interpretation of data for the work. Each author had role in drafting the work and revising it critically. Each author gave final approval of the version to be published and they agree to be accountable for all aspects of the work in ensuring that questions related to the accuracy or integrity of any part of the work are appropriately investigated and resolved.

Conflict of Interest: None.
